# Optimisation of GaN LEDs and the reduction of efficiency droop using active machine learning

**DOI:** 10.1038/srep24862

**Published:** 2016-04-26

**Authors:** Bertrand Rouet-Leduc, Kipton Barros, Turab Lookman, Colin J. Humphreys

**Affiliations:** 1 Department of Materials Science and Metallurgy, University of Cambridge, Cambridge CB3 0FS, UK; 2 Theoretical Division, Los Alamos National Laboratory, Los Alamos, NM 87545, USA

## Abstract

A fundamental challenge in the design of LEDs is to maximise electro-luminescence efficiency at high current densities. We simulate GaN-based LED structures that delay the onset of efficiency droop by spreading carrier concentrations evenly across the active region. Statistical analysis and machine learning effectively guide the selection of the next LED structure to be examined based upon its expected efficiency as well as model uncertainty. This active learning strategy rapidly constructs a model that predicts Poisson-Schrödinger simulations of devices, and that simultaneously produces structures with higher simulated efficiencies.

The use of GaN-based light emitting diodes (LEDs) for very high light output applications is limited by their so-called efficiency droop^1^. Beyond high current densities (>10 A/cm^2^), the efficiency of LEDs at room temperature quickly drops as a function of injected current. There is still an ongoing debate as to the causes of this droop, the main proposed mechanisms being Auger recombination^2^,^3^, hole injection efficiency^4^,^5^, carrier escape from the active region^6^, and carrier delocalisation effects^7^,^8^,^9^. However, the various models link the droop mechanism to high carrier concentrations within the active region of the LED^10^,^11^. In this regard, to delay the onset of droop with respect to the injected current, one seeks optimised LED structures such that carrier concentrations are spread out evenly across the active region. Although the Poisson-Schrödinger simulations presented here are not fully accurate, the same optimisation strategy will also apply to laboratory fabrication of LED structures. Our approach allows us to rapidly construct a model that maps LED structure to simulated efficiency, thereby overcoming time-consuming trial and error based simulations. The simulated efficiencies at high current densities of our machine-learning optimised structures exceed those of reference LED structures by close to 40%.

Our strategy is to use statistical and machine learning (ML) techniques to accelerate the LED design process by suggesting new structures to sample (i.e. build or simulate) in a highly targeted way. We leverage the Efficient Global Optimisation (EGO) strategy of Jones *et al.*^12^, which iteratively selects sample points to maximise expected efficiency improvement while simultaneously accounting for model uncertainty. Provided with a “database” of LEDs with known structure (i.e. number of layers, their composition, doping, and widths) and labelled by resulting electro-luminescence internal quantum efficiency, we build an ML regression model to make predictions of the efficiency for as-yet unseen structures. After each new structure is sampled it is added to the database and the ML model is updated. In a greedy approach to experimental design, one samples new LED structures for which the ML model predicts greatest efficiency. However, to find a globally optimum LED, it turns out to be better to strike a more delicate balance between exploration and exploitation. In selecting new sample points, we should also favor structures for which the ML model is uncertain, to increase the chance of serendipitous discovery. This tradeoff serves to improve the *global* accuracy of the ML model, and thereby minimises the chance of getting stuck in a region of the LED design space for which the efficiencies are only local maxima.

## Global Optimisation using Gaussian Processes

There are two components in our approach to optimisation: The use of Gaussian process (GP) regression to predict LED efficiency, and the EGO heuristic to select the next sample point that maximises the *expected improvement* in efficiency according to the GP model uncertainty. The use of GPs as a predictive tool started in the 1940’s with the basic theory introduced by Kolmogorov and Wiener^13^. In the geosciences, GP regression is known as kriging^14^. Our work builds upon a long history of applying GP to emulate computer simulations^15^,^16^ and as a component of global optimisation^12^,^14^,^17^,^18^,^19^,^20^. A key advantage of GP regression is that it includes *uncertainties* along with its predictions. For a review of GPs in the context of ML see ref. 21.

A GP may be thought of as an infinite-dimensional generalization of a multivariate normal distribution. In our application, we work in the space of LED structures **x** and the GP is used to model the probability distribution of the simulated electro-luminescence efficiency, *y* = *f*(**x**). GPs can be given a Bayesian interpretation in which the general knowledge of the function being learned (such as smoothness and variation length-scale) is modeled as a ‘prior’ distribution over the general space of functions. Then, given a dataset 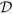 of LED structures and their efficiencies, one uses Bayes rule to obtain a ‘posterior’ normal distribution for LED efficiency^21^. Specifically, for each as-yet unobserved LED structure **x**, the GP model produces a posterior distribution 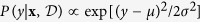, with *μ* the expected efficiency and *σ* its standard deviation. In this study, we use the gaussian_process module of the scikit-learn Python package^22^,^23^. We choose a squared-exponential auto-correlation function and hyper-parameters determined by the maximum likelihood principle.

Given a dataset 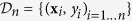 of LED structures and their measured efficiencies, the GP model produces a normal posterior distribution 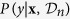 with mean efficiency *μ* and standard deviation *σ* for the structure **x**. The remaining question concerns experimental design: what is the best strategy to select new LED structures 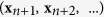 such that we most quickly find LEDs with very high efficiencies? The EGO method provides a simple approximate answer: at each step in the design loop, the next LED structure to sample should be selected to *optimise the expected improvement in efficiency*, after accounting for model uncertainty^12^. Concretely, let *y*_max_ denote the efficiency of the best LED device currently in our dataset 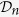. The expected efficiency improvement may be expressed as:


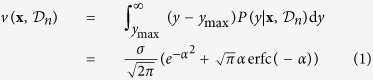


where erfc (·) denotes the complementary error function and 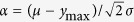 is the scaled difference between the expected efficiency of **x** and the best LED in our dataset.

We select the next sample point to optimise this objective function,


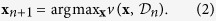


The new LED structure **x**_*n*+1_ becomes an input to a Poisson-Schrödinger code, described below, which calculates the simulated efficiency *y*_*n*+1_. Next, we extend our dataset, 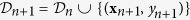 and update the GP posterior, 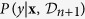, from which we can select *another* sample point via Eq. (2). This iterative process is repeated until a satisfactory LED structure is found. Simultaneously, we obtain a predictive ML model of LED efficiency over a broad range of inputs.

To better understand the objective function in Eq. (1), we evaluate it in two asymptotic limits. In the limit of vanishing uncertainty, σ → 0 (equivalently *α* → ±∞), we observe 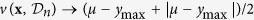. That is, when the model is very certain, the objective function seeks primarily to select points **x** with expected efficiency *μ* better than the best known, *y*_max_. Conversely, imagine that the model uncertainties *σ* are relatively large compared to *μ* − *y*_max_. In this *α* → 0 limit we observe 
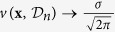
. Thus, when there is no obvious opportunity to improve on the best known LED, the learning strategy becomes primarily exploratory and favors points **x** with the largest model uncertainty. For intermediate *α* ~ 1 the strategy of Eq. (2) balances exploitation (maximizing *μ*) and exploration (maximizing *σ*). In this way, we avoid getting stuck in local maxima: once a region of very efficient LEDs has been well explored, the algorithm samples from a region of larger uncertainty, even if the predicted efficiency is not great.

## Automated LED Design

**Figure 1:**
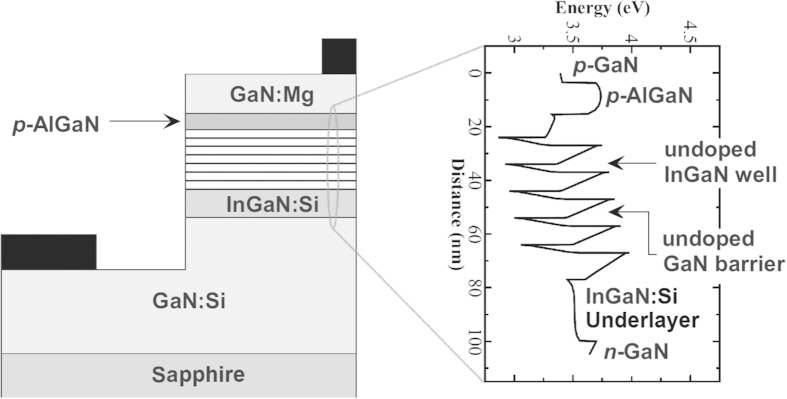
Schematic of the LED that the learning algorithm was given the task to optimise. Left: reference LED structure. Right: conduction band of the active region of the reference LED structure, at high current density (75 A/cm^2^).

In this work, we take the point **x** to represent the structure of the 5-well active region in a GaN-based LED (see Fig. 1 for a schematic). Each input point **x** has 6 parameters: the indium composition of each quantum well and the collective indium composition of the quantum barriers. The quantum well width varies with the indium composition of both well and barrier to keep the wavelength approximately constant. To determine the simulated efficiency of each structure, we use the APSYS software package with materials parameters taken from^24^ and current density 75 A/cm^2^. The band structure was calculated using the 6 × 6 k.p method^25^ in a finite volume approximation. The carrier transport equations were self-consistently computed and coupled with Schrödinger’s equation to determine the confined states in the QWs. Schrödinger and Poisson equations are solved iteratively to account for the band structure deformation with carrier redistribution. The carrier transport consists of drift-diffusion of electrons and holes, Fermi statistics, and thermionic emission at hetero-interfaces, as well as band-to-band tunneling.

**Figure 2:**
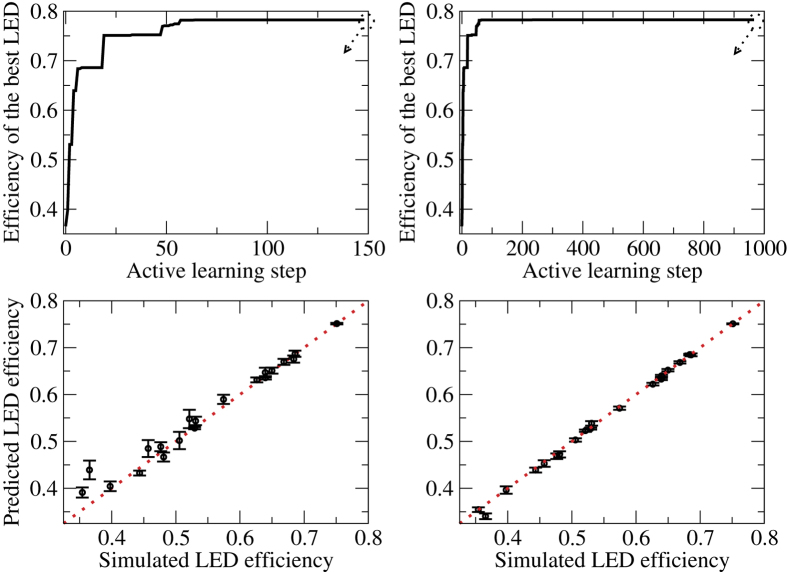
Top row: Simulated room temperature EL internal quantum efficiency of the best LED structure known at each learning step. Bottom row: Efficiencies predicted by the machine learning algorithm versus simulated efficiencies for structures unseen by the algorithm (out of sample) after 150 (left) and 1000 (right) iterations. The error bars indicate the uncertainty of the machine learning model.

We use the machine learning algorithm in Eqs. (1) and (2) to optimise the internal quantum efficiency within the 6-dimensional space (the In content of each the 5 wells and the average In content of the barriers) of our LED structures. As can be seen in Fig. 2a, the procedure converges rapidly, finding a nearly optimal simulated LED efficiency in about 75 iterations. Subsequent iterations make little improvement upon optimal LED efficiency (Fig. 2b), and instead focus on decreasing model uncertainty. Between learning steps 150 through 1000 (Fig. 2c,d), this procedure constructs a very robust model over the global space of LED structures. At iteration 1000 the algorithm is fully converged, and the coefficient of determination is *R*^2^ > 0.99, as determined by cross-validation.

**Figure 3:**
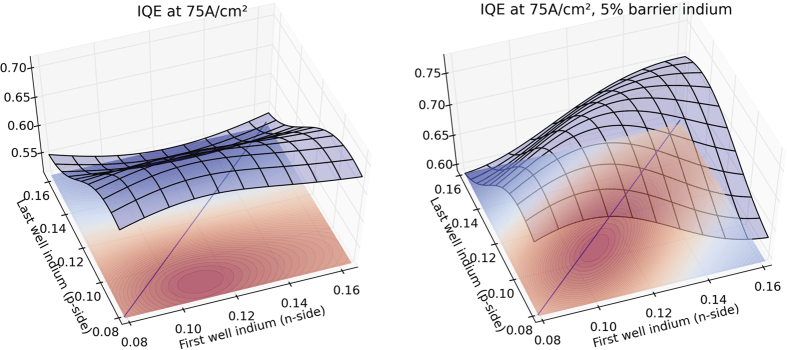
Gaussian process model of expected LED efficiency as a function of the indium content of the individual wells in the active region, linearly interpolated from the n-side (first well) to the p-side (last well). The model is built upon 1000 APSYS simulations spawned by the active learning algorithm and predicts LED efficiency with near perfect accuracy. Left: No indium in the barriers. Right: 5% indium in the barriers. In both cases the optimum is reached with the indium content of the wells decreasing from the n-side to the p-side.

The very high accuracy model provides also some physical insight into the Poisson-Schrodinger simulations. While the drift-diffusion model predicts that most of the light emission of a standard LED structure comes from the 2 top wells, in agreement with electro-luminescence experiments^26^, it also informs us that allowing the indium content of the individual wells to vary across the active region increases the carrier and light emission spreading, in agreement with recent electro-luminescence experiments^27^. As can be seen in Fig. 3, our active learning algorithm finds several optima, which have in common a diminishing indium content in the quantum wells from the n-side to the p-side and the use of InGaN barriers rather than GaN barriers. The diminishing indium content reduces the confinement in the p-side wells^28^, which otherwise concentrate most carriers. The diminishing indium content and the use of InGaN quantum barriers increases the thermionic emission and tunnelling through the hetero-interfaces^29^, allowing the carriers to spread more easily across the active region.

**Figure 4:**
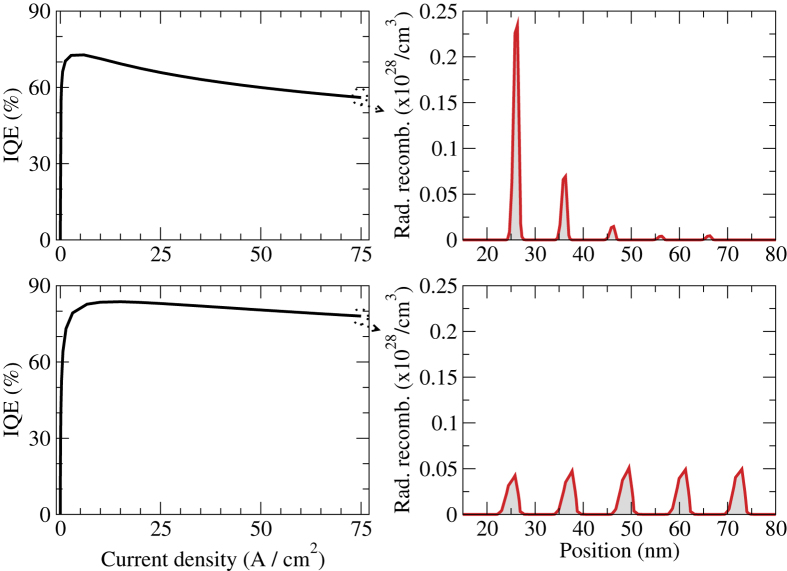
Comparison between the simulation of an initial LED structure (top row) with the simulation of an LED structure optimised by the machine learning algorithm (bottom row). Left: EL Internal quantum efficiency (IQE) as a function of current density. Right: Radiative recombination rate through the active region, at a current density of 75 A/cm^2^. The p-side is towards the left. The optimised structure has wider wells, that are getting shallower (less In) towards the p-side.

The decreasing indium content with increasing well number is associated with increasing well widths for a constant peak emission wavelength. At high current, Auger recombination grows more rapidly with carrier concentration than radiative recombination, and wider wells that compensate for the low indium content become beneficial^30^, as the carrier spreading within each well is increased. Figure 4 draws a comparison between the simulation of a standard LED structure that has GaN barriers and identical wells with the simulation of a machine learning optimised LED structure. The optimised structure achieves an increased spreading of the radiative recombination events: within the wells due to wider wells, which should be beneficial at high currents^31^, and between the wells due to a high barrier indium content and a decreasing indium content towards the p-side of the active region.

To summarize, our active learning strategy rapidly finds LED structures with nearly optimal quantum efficiency while simultaneously building a GP regression model that is predictive for a wide range of LEDs. We used the objective function in (1) for experimental design, which balances the trade-off between exploitation (high predicted efficiency) and exploration (high model uncertainty). At each iteration in our algorithm, the objective function guides the selection of a new LED structure which we simulate, and then use to expand our GP model.

Interestingly, this automated approach finds LEDs that a human expert would strive to design: a structure that spreads evenly the carrier recombination events through the active region of the LED, maximising the radiative recombination events. Leaving the algorithm to optimise the indium content of the active region, we find much higher simulated efficiencies than in standard LEDs. This structure employs a high barrier indium content and a decreasing well indium content towards the p-side of the active region to prevent the accumulation of carriers on the p-side and improve the spreading of the carriers and the radiative recombination events. It also employs wider wells to compensate for wavelength changes with indium content, and to achieve a carrier spreading within the quantum wells that is desirable at high currents.

Our modelling of gallium nitride devices with Poisson-Schrödinger solvers provides qualitative information rather than quantitative predictions. Nevertheless, the algorithm we present demonstrates the power of machine learning for device design. Our method also applies to the optimisation of different LED structures than those presented here. When used in conjunction with actual materials fabrication, our method readily extends to the design of experimental devices. This work is currently ongoing.

## Conclusions

Materials informatics is an emerging field^32^,^33^,^34^ with great promise for functional materials design^35^,^36^,^37^,^38^,^39^. This approach has not yet been adopted by the LED community, despite great potential for improving physical understanding and for accelerating structural design of devices. In this work, we demonstrate that active learning based global optimisation can rapidly and automatically explore Poisson-Schrödinger simulations of gallium nitride devices, and can accelerate the discovery of efficient LEDs. We are currently using this machine-learning approach to guide the growth of experimental structures.

## Additional Information

**How to cite this article**: Rouet-Leduc, B. *et al.* Optimisation of GaN LEDs and the reduction of efficiency droop using active machine learning. *Sci. Rep.* **6**, 24862; doi: 10.1038/srep24862 (2016).

## References

[b1] Stevenson R. The LED’s dark secret. Spectrum, IEEE 46, 26–31 (2009).

[b2] Gardner N. F. *et al.* Blue-emitting InGaN-GaN double-heterostructure light-emitting diodes reaching maximum quantum efficiency above 200A/cm2. Applied Physics Letters 91, 243506 (2007).

[b3] Iveland J., Martinelli L., Peretti J., Speck J. S. & Weisbuch C. Direct measurement of Auger electrons emitted from a semiconductor light-emitting diode under electrical injection: Identification of the dominant mechanism for efficiency droop. Phys. Rev. Lett. 110, 177406 (2013). 23679777 10.1103/PhysRevLett.110.177406

[b4] Xie J. *et al.* On the efficiency droop in InGaN multiple quantum well blue light emitting diodes and its reduction with p-doped quantum well barriers. Applied Physics Letters 93, 121107 (2008).

[b5] Pope I. A. *et al.* Carrier leakage in InGaN quantum well light-emitting diodes emitting at 480 nm. Applied Physics Letters 82, 2755–2757 (2003).

[b6] Schubert M. F. *et al.* On resonant optical excitation and carrier escape in GaInN/GaN quantum wells. Applied Physics Letters 94, 081114 (2009).

[b7] Hangleiter A. *et al.* Anti-localization suppresses non-radiative recombination in GaInN/GaN quantum wells. Philosophical Magazine 87, 2041–2065 (2007).

[b8] Oliver R. A. *et al.* Microstructural origins of localization in InGaN quantum wells. Journal of Physics D: Applied Physics 43, 354003 (2010).

[b9] Badcock T. J. *et al.* Carrier density dependent localization and consequences for efficiency droop in InGaN/GaN quantum well structures. Japanese Journal of Applied Physics 52, 08JK10 (2013).

[b10] Laubsch A. *et al.* On the origin of IQE-droop in InGaN LEDs. physica status solidi (c) 6, S913–S916 (2009).

[b11] Lin G.-B. *et al.* Analytic model for the efficiency droop in semiconductors with asymmetric carrier-transport properties based on drift-induced reduction of injection efficiency. Applied Physics Letters 100, 161106–161106-4 (2012).

[b12] Jones D. R., Schonlau M. & Welch W. J. Efficient global optimization of expensive black-box functions. Journal of Global optimization 13, 455–492 (1998).

[b13] Wiener N. Extrapolation, interpolation, and smoothing of stationary time series, vol. 2 (MIT press Cambridge, MA, 1949).

[b14] Journel A. & Huijbregts C. J. Mining Geostatistics (Academic Press, London, 1978).

[b15] O’Hagan A. & Kingman J. Curve fitting and optimal design for prediction. Journal of the Royal Statistical Society. Series B (Methodological) 40, 1–42 (1978).

[b16] Sacks J., Welch W. J., Mitchell T. J. & Wynn H. P. Design and analysis of computer experiments. Statistical Science 4, 409–423 (1989).

[b17] Gupta S. S. & Miescke K. J. Bayesian look ahead one-stage sampling allocations for selection of the best population. Journal of Statistical Planning and Inference 54, 229–244 (1996). 40 Years of Statistical Selection Theory, Part I.

[b18] Osborne M. A., Garnett R. & Roberts S. J. Gaussian processes for global optimization. In *3rd international conference on learning and intelligent optimization (LION3),* 1–15 (Trento, Italy, January 14–18, 2009).

[b19] Frazier P., Powell W. & Dayanik S. The knowledge-gradient policy for correlated normal beliefs. INFORMS Journal on Computing 21, 599–613 (2009).

[b20] Srinivas N., Krause A., Kakade S. & Seeger M. Information-theoretic regret bounds for gaussian process optimization in the bandit setting. Information Theory, IEEE Transactions on 58, 3250–3265 (2012).

[b21] Rasmussen C. E. Gaussian processes for machine learning (MIT Press, 2006).

[b22] Pedregosa F. *et al.* Scikit-learn: Machine learning in python. J. Mach. Learn. Res. 12, 2825–2830 (2011).

[b23] Lophaven S. N., Nielsen H. B. & Søndergaard J. Dace-a matlab kriging toolbox, version 2.0. Technical Report IMM-REP-2002-1. (IMM Technical University of Denmark, Lyngby, Denmark, 2002).

[b24] Piprek J. Nitride semiconductor devices: principles and simulation (John Wiley & Sons, 2007).

[b25] Chuang S. L. Efficient band-structure calculations of strained quantum wells. Phys. Rev. B 43, 9649–9661 (1991). 10.1103/physrevb.43.9649 9996663

[b26] David A. *et al.* Carrier distribution in (0001)InGaN-GaN multiple quantum well light-emitting diodes. Applied Physics Letters 92 (2008).

[b27] Kang D. *et al.* Improving output power performance of InGaN-based light-emitting diodes by employing step-down indium contents. Japanese Journal of Applied Physics 54, 042102 (2015).

[b28] Sze S. M. & Ng K. K. Physics of semiconductor devices (John Wiley & Sons, 2006).

[b29] Grinberg A. A., Shur M., Fischer R. & Morkoc H. An investigation of the effect of graded layers and tunneling on the performance of AlGaAs/GaAs heterojunction bipolar transistors. Electron Devices, IEEE Transactions on 31, 1758–1765 (1984).

[b30] Li X. *et al.* Impact of active layer design on InGaN radiative recombination coefficient and LED performance. Journal of Applied Physics 111, 063112 (2012).

[b31] Ren C. X. Polarisation fields in III-nitrides: effects and control. Materials Science and Technology 0, 1–16 (2015).

[b32] Rajan K. Materials informatics. Materials Today 8, 38–45 (2005).

[b33] Jain A. *et al.* Commentary: The materials project: A materials genome approach to accelerating materials innovation. APL Materials 1, 011002 (2013).

[b34] Balachandran P. V., Theiler J., Rondinelli J. M. & Lookman T. Materials prediction via classification learning. Scientific reports 5, 13285 (2015). 26304800 10.1038/srep13285 PMC4548442

[b35] Bhadeshia H. K. D. H. Neural networks in materials science. ISIJ International 39, 966–979 (1999).

[b36] Hautier G., Fischer C. C., Jain A., Mueller T. & Ceder G. Finding nature’s missing ternary oxide compounds using machine learning and density functional theory. Chemistry of Materials 22, 3762–3767 (2010).

[b37] Pilania G., Wang C., Jiang X., Rajasekaran S. & Ramprasad R. Accelerating materials property predictions using machine learning. Scientific Reports 3, 2810 (2013). 24077117 10.1038/srep02810 PMC3786293

[b38] Castelli I. E. & Jacobsen K. W. Designing rules and probabilistic weighting for fast materials discovery in the perovskite structure. Modelling and Simulation in Materials Science and Engineering 22, 055007 (2014).

[b39] Ghiringhelli L. M., Vybiral J., Levchenko S. V., Draxl C. & Scheffler M. Big data of materials science: Critical role of the descriptor. Physical Review Letters 114, 105503 (2015). 25815947 10.1103/PhysRevLett.114.105503

